# Analysing the Cyanobacterial PipX Interaction Network Using NanoBiT Complementation in *Synechococcus elongatus* PCC7942

**DOI:** 10.3390/ijms25094702

**Published:** 2024-04-25

**Authors:** Carmen Jerez, Antonio Llop, Paloma Salinas, Sirine Bibak, Karl Forchhammer, Asunción Contreras

**Affiliations:** 1 Departamento de Fisiología, Genética y Microbiología, Universidad de Alicante, 03690 San Vicente del Raspeig, Spain; carmen.jerez@ua.es (C.J.); antonio.llop@ua.es (A.L.); paloma.salinas@ua.es (P.S.); sb221@alu.ua.es (S.B.); 2 Interfaculty Institute of Microbiology and Infection Biology, University Tübingen, 72076 Tübingen, Germany; karl.forchhammer@uni-tuebingen.de

**Keywords:** NanoLuc, PII, NtcA, nitrogen regulation, energy regulation, protein-fragment complementation assays, PCAs, complementation reporter, environmental factors, 2-oxoglutarate

## Abstract

The conserved cyanobacterial protein PipX is part of a complex interaction network with regulators involved in essential processes that include metabolic homeostasis and ribosome assembly. Because PipX interactions depend on the relative levels of their different partners and of the effector molecules binding to them, in vivo studies are required to understand the physiological significance and contribution of environmental factors to the regulation of PipX complexes. Here, we have used the NanoBiT complementation system to analyse the regulation of complex formation in *Synechococcus elongatus* PCC 7942 between PipX and each of its two best-characterized partners, PII and NtcA. Our results confirm previous in vitro analyses on the regulation of PipX-PII and PipX-NtcA complexes by 2-oxoglutarate and on the regulation of PipX-PII by the ATP/ADP ratio, showing the disruption of PipX-NtcA complexes due to increased levels of ADP-bound PII in *Synechococcus elongatus*. The demonstration of a positive role of PII on PipX-NtcA complexes during their initial response to nitrogen starvation or the impact of a PipX point mutation on the activity of PipX-PII and PipX-NtcA reporters are further indications of the sensitivity of the system. This study reveals additional regulatory complexities in the PipX interaction network, opening a path for future research on cyanobacteria.

## 1. Introduction

Cyanobacteria, phototrophic prokaryotes that perform oxygenic photosynthesis, constitute an ecologically important phylum that is responsible for the evolution of the oxygenic atmosphere and are the main contributors to marine primary production [1,2]. In addition, their photosynthetic lifestyle and ease of cultivation make them ideal production systems for a number of high-value compounds, including biofuels [3]. Cyanobacteria have developed sophisticated regulatory systems to adapt to the challenging environmental conditions that they face [4,5].

The cyanobacterium *Synechococcus elongatus* PCC 7942 (hereafter *S. elongatus*) is a model system used to address fundamental questions concerning the phylum’s photosynthetic lifestyle. *S. elongatus* is, so far, the only photosynthetic organism for which the contribution of each gene to its fitness has been evaluated [6,7]. Despite important breakthroughs in the genetic analysis of cyanobacteria, there are still remarkable proportions of genes of unknown functions and of unique genes, many of which are presumably involved in functions relevant to the biology of cyanobacteria. One of these unique cyanobacterial proteins is PipX (PII interacting protein X), identified by its ability to form complexes with PII (encoded by *glnB*) and NtcA [8,9], two 2-oxoglutarate (2-OG) sensors which have critical roles in carbon/nitrogen homeostasis [10,11,12,13].

PII regulates the activity of the proteins involved in nitrogen and carbon metabolism in bacteria and plants through direct protein–protein interactions [11] and perceiving metabolic information through the competitive binding of ATP or ADP and the synergistic binding of ATP and 2-OG [14,15,16]. The first PII targets identified in cyanobacteria were NAGK (N-Acetyl Glutamate Kinase) and PipX, detected by yeast two-hybrid approaches in *S. elongatus* [8,9,17,18]. Their interactions with PII have recently been quantified in vitro, with unprecedented sensitivity, using Split NanoBiT technology [19].

In response to nitrogen limitations, PipX coactivates the regulon of the cyanobacterial global transcriptional regulator NtcA [20,21,22,23,24,25]. The PipX-NtcA complex consists of one active (2-OG-bound) NtcA dimer and two PipX molecules, each binding to a NtcA subunit [26]. PipX stabilizes the conformation of NtcA which is transcriptionally active and probably helps local recruitment of RNA polymerase. The binding of PipX to PII or NtcA is antagonistically tuned by 2-OG levels; while high levels of 2-OG favour the interaction of PipX with NtcA, they prevent the PipX-PII interactions [8,9,26,27].

PipX uses the same surface to bind to either 2-OG-bound NtcA, stimulating DNA binding and transcriptional activity, or to 2-OG-free PII. The PII sequestration of PipX at low 2-OG renders PipX unavailable for NtcA binding and activation, reducing the expression of NtcA-dependent gene targets [26,27,28,29,30,31,32]. In addition, the interaction between PII and PipX is highly sensitive to fluctuations in the ATP/ADP ratio, and thus the energy state of the cells [33,34].

Different studies have suggested that PipX forms part of an extended regulatory network beyond PII and NtcA [35,36,37,38,39]. PlmA, a transcriptional regulator found exclusively in cyanobacteria, was identified as a member of the PipX interaction network by yeast three-hybrid searches using PipX-PII as bait [37]. Gradient profiling by sequencing (Grad-seq) showed that PipX co-localizes with either the metabolic regulators PII, NtcA and PlmA or with the RNA–protein complexes involved in transcription, RNA metabolism and translation initiation [40]. A synteny analysis in cyanobacteria connected PipX with additional proteins [39], one of which was the ribosome-assembly GTPase EngA, whose binding to PipX and regulatory connections in *S. elongatus* have already been demonstrated [36,41].

The regulatory complexity of the PipX interaction network challenges investigations into its physiological significance and the contribution of environmental factors to the formation and regulation of the different PipX complexes. Complex formation depends on the relative levels of the different PipX partners and of the effector molecules that may bind to them [42], but these are not always known. In this intricate context, keeping the intracellular environment as untouched as possible should help to address the relevant questions about the PipX interaction network.

The NanoBiT complementation system [43], based on the reconstitution of the small- and high-output bioluminescence enzyme NanoLuc, has been used in both mammalian [44,45,46] and bacterial [47,48,49] cells to demonstrate the specificity of protein interactions in their natural environment. Very recently, it has been used to study the effect of metabolic fluctuations on interactions mediated by the cyanobacterial PII protein in an *E. coli* host [49]. However, detailed studies of the regulation of complex formation in model systems have not been reported.

Here, we use the NanoBiT complementation system to analyse the regulation of complex formation within the *S. elongatus* interaction network. Our results, in full agreement with the information generated by previous analyses of PipX-PII or PipX-NtcA interactions, reveal additional regulatory complexity. This work constitutes a breakthrough in the field of the signalling and interaction networks in cyanobacteria.

## 2. Results and Discussion

### 2.1. Reporter Constructs and Strains Used to Analyse PipX-PII and PipX-NtcA Interactions in S. elongatus

**Figure 1:**
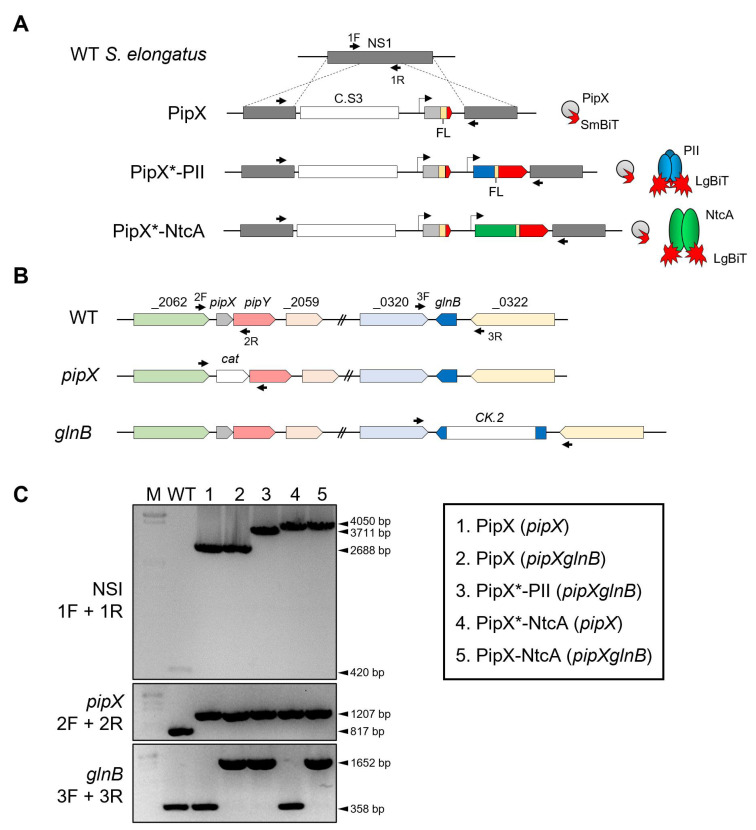
NanoBiT constructs and strategy used to analyse the PipX-PII and PipX-NtcA interactions in *S. elongatus*. (**A**) The NSI region and derivatives containing the C.S3 selection marker and the corresponding gene fusions are schematically illustrated, with the relevant products depicted to the right. * refers to PipX or PipX^Y6A^. (**B**) Schematic representation of the *pipX* and *glnB* alleles. (**C**) **Left** panel: PCR analysis indicating the primers, depicted as black arrows, in (**A**,**B**) and the size of bands at the left and right, respectively. M: λ EcoRI/HindIII size marker. **Right** panel: strains analysed. See text for additional details.

The constructs used here to co-express the NanoBiT-based fusion proteins in *S. elongatus* and additional controls are shown in Figure 1.

The design of protein fusions was guided by previously validated NanoBiT fusions for in vitro assays using the PipX or PII proteins from *Synechocystis* sp. PCC6803 [19]. PipX was fused to the SmBiT fragment (PipX-SmBiT), while PII and NtcA were fused to the LgBiT fragment (PII-LgBiT or NtcA-LgBiT). Flexible linkers of 16 or 8 amino acids, respectively, were introduced at the C-terminus of PipX or of PII and NtcA. A total of three NSI-derivative insertions containing PipX-SmBiT fusions alone, or in combination with PII-LgBiT or NtcA-LgBiT, were generated. To allow for physiological control of the levels of the interacting proteins during environmental changes, the *pipX, glnB* or *ntcA* derivatives were expressed from their corresponding promoter. To facilitate the introduction of reporters into the neutral site I (NSI) of the *S. elongatus* chromosome by allelic replacement, a Streptomycin-resistant marker cassette (C.S3) was included within the NSI insertions. The NSI-carrying plasmid pUAGC280 was used as a vector to obtain derivatives containing the relevant insertions, generating plasmids pUAGC1160, pUAGC1161 and pUAGC1163 (Table 1). For simplicity, we will refer hereafter to the NSI derivatives as PipX, PipX-PII or PipX-NtcA constructs or, more specifically, as the PipX control and PipX-PII or PipX-NtcA reporters.

To analyse the impact of a PipX point mutation (Y6A), known to impair contact between PII and NtcA [26], PipX^Y6A^-PII and PipX^Y6A^-NtcA reporters were also generated. The plasmids pUAGC1161 and pUAGC1163 were used to obtain, respectively, the plasmids pUAGC1162 and pUAGC1164 (Table 1).

To minimise the interference of endogenous proteins with the activity of the NanoBiT reporters and unwanted recombination events in *S. elongatus*, *pipX* and *pipXglnB* null derivatives of *S. elongatus* were chosen as host strains for the PipX or PipX-NtcA constructs and the PipX-PII reporter, respectively (Table 2). Although *glnB* is essential in *S. elongatus*, it can be inactivated in a *pipX* background [28,29,31] and, importantly, the *pipX* and *pipXglnB* null mutants show no significant growth defects under constant standard culture conditions [33,50]. In contrast, *ntcA* is essential in *S. elongatus* in both wild-type [6,51] and *pipX* backgrounds, prompting us to use the *pipX* mutant as the default strain for PipX-NtcA reporter analyses. It is worth noting that the potential for interference is greatest for PII due to its abundance [52], its high affinity for PipX [8,26,28,29,31,33,34] and its trimeric structure, circumstances that would allow it to bind to PipX-SmBiT and/or PII-LgBiT, to the detriment of the luciferase signal.

**Table 1:** Plasmids.

Plasmid	Description, Relevant Characteristics	Reference or Source
pUAGC280	*P_trc_* into *NSI*, Ap^R^ Sm^R^	[53]
pUAGC1160	(*P_pipX_:pipX:FL:SmBiT*) into *NSI*, Ap^R^ Sm^R^	This work
pUAGC1161	(*P_pipX_:pipX:FL:SmBiT P_glnB_:glnB:FL:LgBiT*) into *NSI*, Ap^R^ Sm^R^	This work
pUAGC1162	(*P_pipX_:pipX^16ta>gc^:FL:SmBiT P_glnB_:glnB:FL:LgBiT*) into *NSI*, Ap^R^ Sm^R^	This work
pUAGC1163	(*P_pipX_:pipX:FL:SmBiT P_ntcA_:ntcA:FL:LgBiT*) into *NSI*, Ap^R^ Sm^R^	This work
pUAGC1164	(*P_pipX_:pipX^16ta>gc^:FL:SmBiT P_ntcA_:ntcA:FL:LgBiT*) into *NSI*, Ap^R^ Sm^R^	This work
PII-ST-FL-LgBiT	P*_terR_:*PII-StrepTag-FL-LgBiT	[19]
pUAGC126	*pipX* replaced with *cat*, Ap^R^ Cm^R^	[38]
pPM128	*CK2* (+) into *glnB*, Km^R^	[54]

Km, kanamycin. Sm, streptomycin. ^R^, resistance. Cm, chloramphenicol. cat, chloramphenicol acetyltransferase.

A total of seven strains combining one NanoBiT construct at their NSI site with the inactivation alleles for *pipX* or *pipX* and *glnB* at their original loci were generated during this study (Table 2). In each case, independent streptomycin-resistant clones from each transformation were PCR-analysed to confirm the complete segregation of the modified NSI alleles in *S. elongatus* and three validated clones were selected for additional analysis. For simplicity, the PCR analyses carried out to verify the construction of the strains used in this work have been combined in Figure 1C, and those from Y6A-derivative strains, indistinguishable from their wild-type counterparts, have been omitted.

### 2.2. PII-LgBiT and PipX-SmBiT Retain Their Regulatory Features in S. elongatus

Since PipX’s levels in *S. elongatus* are impaired by mutations decreasing its binding to PII or by environmental conditions disrupting PipX-PII complexes [28,31,56], the prediction is that, in the absence of PII, the levels of PipX would be significantly impaired. To test this idea, while seeking evidence that our NanoBiT derivatives maintain their regulatory features, we next asked whether the presence of either PII or PII-LgBiT have a positive effect on the levels of PipX-SmBiT.

**Figure 2:**
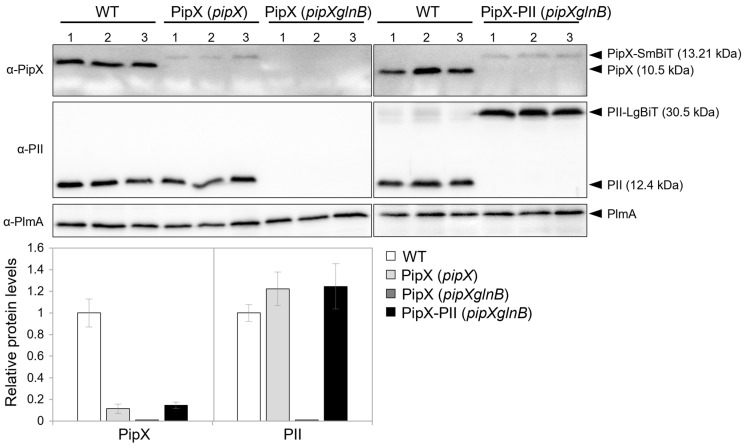
Levels of PipX and PII derivatives in *S. elongatus*. Representative immunodetection of PipX and PII of *S. elongatus* strains differing in their NSI constructs or genetic background (in brackets) as indicated. Relative PipX and PII levels were normalized by the PlmA signal and referred to the WT. Data are presented as means and error bars (standard deviation) from three biological replicates.

Western blots with anti-PipX or anti-PII antibodies were carried out using the PipX control or PipX-PII reporter constructs in *S. elongatus* and its mutant derivatives. As shown in Figure 2, both PipX-SmBiT and PII-LgBiT were detected in the *pipXglnB* double mutant carrying the PipX-PII reporter construct. While PII-LgBiT was detected at roughly the same levels as PII in the WT strain, the signal detected for PipX-SmBiT was about 15% that of PipX in the WT strain, suggesting that the SmBiT fragment decreases the levels of PipX and/or its affinity for the anti-PipX antibody. Importantly, PipX-SmBiT was detected in the *pipX* strain but not in the *pipXglnB* strain, thus providing direct evidence of the importance of PII for PipX levels in *S. elongatus*.

Although we cannot rule out that the SmBiT tag interferes with the recognition of PipX-SmBiT by the anti-PipX antibody, it is reasonable to assume that the SmBiT tag and/or the ectopic location of the *pipX* gene derivative result in comparatively lower levels of PipX-SmBiT in *S. elongatus*. According to this, the PipX-PII reporter strain engineered here would provide informative results with luminescence levels that could potentially be higher.

### 2.3. PipX-PII and PipX-NtcA Reporters Respond in Opposite Ways to a Drop in the Intracellular ATP Levels in S. elongatus

**Figure 3:**
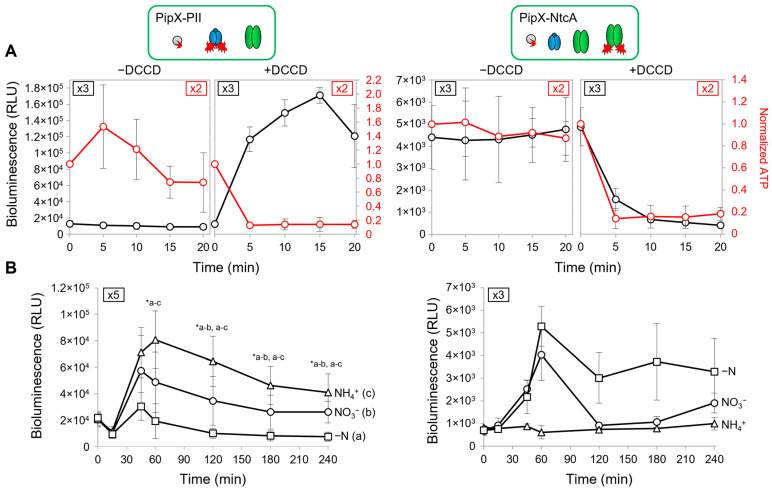
Real-time responses of PipX-PII and PipX-NtcA reporters to changes in energy levels and nitrogen sources. Reporter strains are indicated, and relevant proteins illustrated on top of the corresponding results. (**A**) Bioluminescence signals (black scale and curves) and normalized ATP levels (red scale and curves) from cultures grown with BG11 in the presence or absence of 200 µM DCCD. (**B**) Bioluminescence signals after transfer to the indicated nitrogen regimens. Data in (**A**,**B**) are presented as means with error bars (standard deviation) due to the indicated number of biological replicates (top rectangles) performed in each case. Wilcoxon rank-sum tests between the indicated comparisons produced *p*-values < 0.05 (*). (a–c) refer to the indicated nitrogen conditions.

To test the sensitivity of the NanoBiT PipX-PII and PipX-NtcA reporters to real-time changes in energy levels, the intracellular ATP/ADP ratio was decreased by adding DCCD (N, N-dicyclohexylcarbodiimide), a specific inhibitor of F_o_F_1_-ATP synthase [57], to *S. elongatus* cultures growing in standard nitrate medium. The results are shown in Figure 3A alongside schematic illustrations of the relevant players in the PipX partner swapping in the analysed strains. These carried PipX-PII or PipX-NtcA reporters into *pipXglnB* or *pipX* backgrounds, respectively.

Exponentially growing cultures expressing the PipX-PII reporter in the *pipXglnB* background were divided into two before adding DCCD to half of them to record luminescence and intracellular ATP in real time, at 5 min intervals for up to 20 min (Figure 3A, left). The luminescence values increased (10-fold induction) while a sharp drop in ATP, to approx. 20% of the previous level, took place in the DCCD-containing cultures in less than 5 min after their addition. Since the luminescence and ATP values remained unaltered in the PipX control cultures, the results confirm the sensitivity of the PipX-PII reporter to the intracellular ATP/ADP ratio.

In the case of the PipX-NtcA reporter, the DCCD-containing cultures showed a very fast and strong decrease in luminescence and ATP levels that responded equally to DCCD, dropping to approx. 20% of the previous level (Figure 3A, right). It is worth noting that NtcA does not bind nucleotides and thus the disruption of PipX-NtcA complexes associated with the drop in the ATP/ADP ratio is necessarily indirect. Since it coincides with the drastic increase in luminescence from the PipX-PII reporter, the results reflect strong competition for PII under these conditions, titrating PipX to the detriment of the PipX-NtcA complexes.

In summary, the results shown in Figure 3A clearly reflect the direct, positive and indirect, negative regulations of a low ATP/ADP ratio on PipX-PII and PipX-NtcA complexes, respectively. This is fully consistent with the higher abundance of PII and its very high affinity for PipX, further suggesting that the binding of PipX to its additional targets may also be significantly regulated by the energy levels in *S. elongatus*.

### 2.4. PipX-PII and PipX-NtcA Reporters Respond in Opposite Ways to the 2-OG Levels in S. elongatus

Complex regulation was next analysed under different culture conditions representative of different intracellular levels of the PII effector 2-OG [58,59]. To obtain low, intermedium and high intracellular 2-OG levels we adjusted the nitrogen source, adding, respectively, ammonia, nitrate or no nitrogen source to the culture media. Nitrate cultures growing in an exponential phase were subjected to routine washing protocols before being transferred to fresh media containing either ammonia, nitrate or no added combined nitrogen source. Luminescence was recorded at different timepoints after the media transfer for up to 240 min. The results of this analysis are shown in Figure 3B.

For the PipX-PII reporter, during the first half hour after its transfer to the different media (that is, NH_4_^+^, NO_3_ or -N), its luminescence values remained below the levels of pre-transfer in all three cultures. Its luminescence values significantly increased afterwards, particularly in the ammonium and nitrate cultures, reaching a maximum at the 60′ timepoint, after which they decreased slowly. In contrast, the basal luminescence levels obtained with the PipX control were not affected by the nitrogen source (Figure S1A). Therefore, after an initial period of adaptation (see below), the cultures showed the expected inverse correlation between the inferred intracellular carbon to nitrogen ratio (2-OG levels) and PipX-PII binding.

The luminescence of the PipX-NtcA reporter increased after the 15′ timepoint in nitrate and, to a greater extent, in nitrogen-deprived cultures, with maximal values obtained at around the 60′ timepoint. As expected, while the basal luminescence levels obtained with the PipX control were not affected by the nitrogen source (Figure S1B), reporter cultures showed a direct correlation between nitrogen scarcity (high 2-OG levels associated with the different N regimes) and PipX-NtcA activity, particularly for their maximal luciferase values. In addition, the oscillation of the reporter signal under conditions in which 2-OG accumulates intracellularly (Figure 3B and Figure S2) is compatible with the existence of a negative feedback loop counteracting the overactivation of NtcA by PipX.

The results shown so far (a) indicate that a comparison of the real-time luminescence signals from each of the two reporters in each of the three media generated the expected correlations between reporter activity and intracellular 2-OG levels, (b) confirm the reliability and great sensitivity of the NanoBiT system for comparative in vivo assays and (c) also revealed additional complexities that are further discussed in the following sections.

### 2.5. PipX Levels Decrease in the Absence of Combined Nitrogen in S. elongatus

While the correlations between reporter activity and the intracellular levels of 2-OG shown in Figure 3 reflect the previous knowledge on PipX-PII and PipX-NtcA complexes, the low values of luciferase at the start of the experiment suggested additional regulatory complexity. For instance, the reporter signals were always very low after the transfer of cultures to fresh media, indicating that the culture manipulations performed during the washing protocol clearly disrupted PipX-PII complexes and additionally delayed the formation of new PipX-PII and PipX-NtcA complexes.

Because it is now clear that binding to PII plays a positive role on PipX levels ([56], Figure 2), it appears that the disruption of PipX-PII complexes due to high levels of 2-OG during the washing steps may also impair PipX levels. To test this idea, we performed similar washing steps using either nitrogen-free (BG11_0_) or nitrate-containing (BG11) media in parallel and subsequently determined the levels of PipX in *S. elongatus*. To explore the possible effect of the transfer to fresh media, we also included a control in which the pellets were resuspended in the same BG11 supernatant.

**Figure 4:**
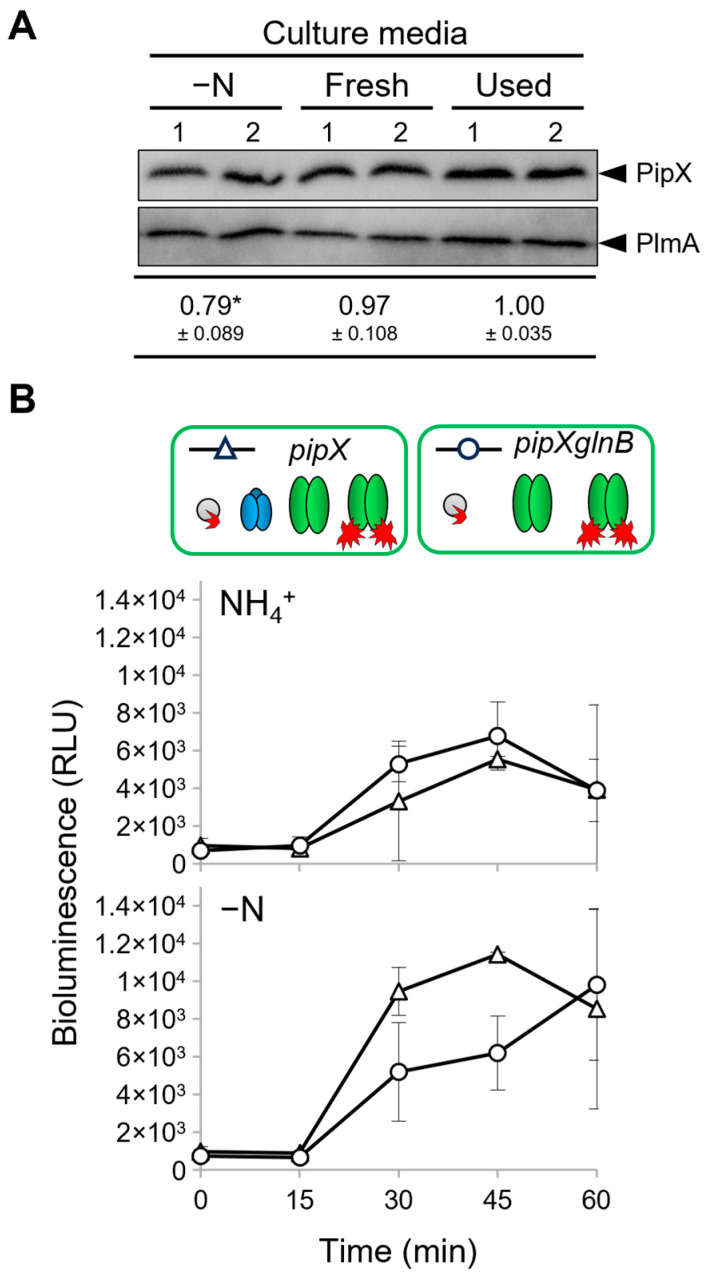
Regulation of PipX levels and PipX-NtcA interactions by PII in response to nitrogen deprivation. (**A**) Representative immunodetection and relative levels of PipX (PipX) from *S. elongatus* cultures after two centrifuge/washing steps with BG11_0_ (-N), BG11 (Fresh) or the same BG11 supernatant (Used), normalised to the intensity shown in the same blot by endogenous PlmA, and respective to the “Used” values. Data are presented as means and error bars (standard deviation) from five biological replicates of two independent experiments. Wilcoxon rank-sum test produced *p*-values < 0.05 (*) (**B**) Real-time comparison of bioluminescence signals under the indicated nitrogen regimens at different times between the *pipX* (Δ) and *pipXglnB* (○) strains. Data are presented as means and error bars (standard deviation) from two biological replicates. Other details as in Figure 3.

As shown in Figure 4A, while using the same or new media during the washing protocol appeared irrelevant to PipX levels, these were significantly lower in the cultures washed with BG11_0_ than in those washed with BG11. Thus, the results further confirm the importance of PipX-PII complexes in maintaining PipX levels in *S. elongatus*.

In summary, the low interaction signals obtained after the transfer of cultures to fresh media and the delay in reaching maximal bioluminescence signals from both PipX-PII and PipX-NtcA reporters is consistent with the requirement of a de novo synthesis of PipX.

### 2.6. The PipX Point Mutation Y6A Drastically Impairs PipX-PII and PipX-NtcA Complexes in S. elongatus

To provide additional evidence of the sensitivity of the NanoBiT constructs used here, we next analysed the impact of the Y6A mutation on reporter activity. Tyr6 is at the PipX surface and is involved in PipX’s contact with PII and NtcA [26], and its mutation to Ala impairs PipX toxicity, NtcA coactivation [31] and PipX levels [56].

**Figure 5:**
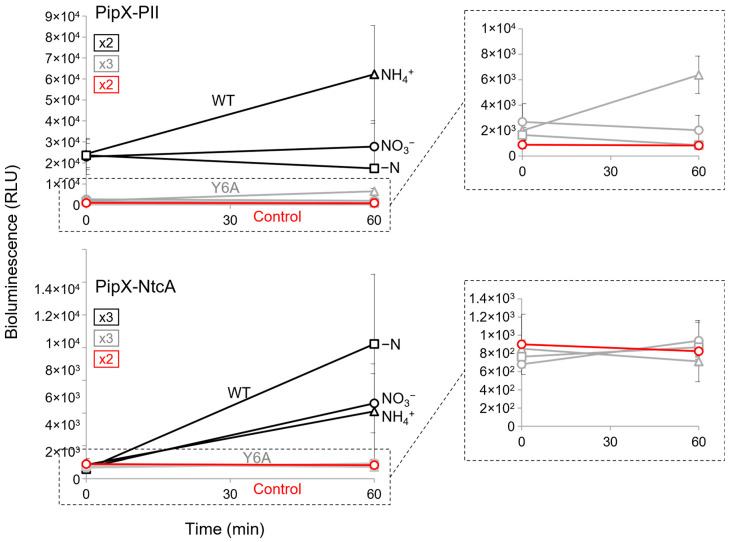
Impact of the mutation Y6A on PipX-PII and PipX-NtcA interactions. Bioluminescence signal of the indicated strains under different nitrogen regimen conditions at timepoints 0 and 60′. Black, grey, and red lines correspond to the WT or Y6A versions of the reporter and to the PipX control, respectively. Data are presented as means and error bars (standard deviation) from the indicated biological replicates (squares inside the graphics). Inset covers enlarge their corresponding regions.

The impact of the Y6A mutation on the activity of the PipX-PII and PipX-NtcA reporters was determined at the timepoints 0 and 60′ after their transfer to ammonia, nitrate or nitrogen-free media. As shown in Figure 5, except for the PipX^Y6A^-PII reporter in ammonium, the levels of the activity of the mutant reporters in any given nitrogen regime were indistinguishable from the basal levels obtained with the PipX control constructs. This rather dramatic impact of the Y6A mutation on the PipX-PII and PipX-NtcA complexes is consistent with the combined effects of impairing both the binding and protein levels of *S. elongatus* and further emphasizes the sensitivity of the NanoBiT system developed here.

### 2.7. PII Plays a Positive Regulatory Role on PipX-NtcA Complexes during Their Initial Response to Nitrogen Deprivation

While it seems clear that, in *S. elongatus*, PII provides strong competition for PipX-NtcA complexes in nitrate, the role of PII at more extreme 2-OG levels is always assumed to be irrelevant or, given the intracellular abundance of PII, slightly negative. However, the drop in PipX levels observed for BG11_0_ cultures (Figure 4A) suggested that PII may play a positive role in PipX-NtcA complexes during their response to nitrogen starvation. To test this idea, we transferred nitrate-cultured cells to media with ammonium or without a nitrogen source and determined the PipX-NtcA reporter activity in *pipX* and *pipXglnB* backgrounds, in parallel.

As shown in Figure 4B, no significant differences in luminescence levels were observed between the *pipX* and *pipXglnB* strains after their transfer into ammonium-containing media, conditions under which luminescence signals are indistinguishable between the PipX-NtcA and PipX constructs in both *pipX* and *pipXglnB* backgrounds. In contrast, the presence of PII in nitrogen-free cultures was associated with higher luminescence levels during the first 15–45 min after the transfer, consistent with a positive, as well as transient, role of PII on PipX-NtcA complex formation under conditions of nitrogen deprivation.

### 2.8. Additional Players May Affect PipX-PII and PipX-NtcA Complexes in S. elongatus

A consequence of the high sensitivity of our NanoBiT reporter analysis, combined with the regulatory complexity and environmental sensitivity of the biological system used, was that the absolute values of the luminescence levels obtained in *S. elongatus* for a given strain and experimental condition varied depending on which of our laboratories the experiment was performed and also between repetitions of the same experiments on different days under apparently identical conditions. In addition, when the bioluminescence levels were just slightly above basal levels, as happened with the PipX-NtcA reporter, when cultures were transferred to ammonium-containing media, the experiments producing lower levels of luminescence were less informative (Figure 3, Figure 4, Figure 5 and Figures S1 and S2). However, despite all this, the tendencies, response to treatments and the differences between the strains being compared was remarkably reproducible. Therefore, rather than integrating data from independent experiments, representative data with several replicates were shown in each of the figures or tables (Tables S2–S5) of this work.

The dynamics of the PipX-PII and PipX-NtcA reporters merit additional comments. The slow decrease in PipX-PII reporter activity after the 60′ timepoint in all three culture conditions, shown in Figure 3, seemed to be independent of the nitrogen source and was not accompanied by a reciprocal increase in PipX-NtcA reporter activity, as would be expected if only PII and NtcA compete for PipX binding. Furthermore, after reaching a maximum at the 60′ timepoint, the PipX-NtcA interaction signal decreased strongly in the nitrate-containing cultures and, given that the 2-OG levels are known to always be higher in nitrate than in ammonium, the phenomenon again appears independent of the nitrogen source, suggesting the involvement of additional regulatory factors. It is thus tempting to propose that a protein binding to PipX, perhaps one of the previously identified components of the PipX interaction or synteny networks, may account for these observations. To address this issue, we will construct NanoBit reporters for the interactions between PipX and candidate proteins from PipX networks.

### 2.9. The NanoBit Approach in the Context of Cyanobacterial Interaction Networks

Popular genetic approaches to protein–protein interactions such yeast or bacterial two-hybrid systems are used to probe the specificity of suspected interactions without considering their regulatory context. Not surprisingly, false negatives are rather frequent in yeast and bacteria two-hybrid systems [60,61,62,63], whose assays are performed in fixed conditions, following standardized protocols [64,65,66,67]. Very much in contrast with this, NanoBit reporters are analysed under chosen conditions in real time and thus give valuable information about the regulation of the corresponding protein complexes by environmental and/or genetic factors, as we have shown here for PipX-PII and PipX-NtcA reporters.

The main limitation of the NanoBit approach is that strain preparation is time-consuming, particularly if the aim is to gain as much information as possible while identifying, avoiding, or minimizing possible artifacts. These might include altered properties or artificial regulations of the protein fusions and interference from unmodified versions of the proteins of interest. Because of this, the comparative simplicity of two-hybrid methods may be better indicated to address specific questions and compare multiple variants of a given protein, as in studies of interaction determinants.

In the context of the cyanobacterial interaction networks exemplified by PipX, the challenge is to show the physiological significance of putative interactions that have been predicted by other methods. In terms of this challenge the NanoBit approach has no rivals.

## 3. Materials and Methods

### 3.1. Plasmid Construction

The plasmids, strains and oligonucleotides used in this work are listed in Table 1, Table 2 and Table S1, respectively. Cloning procedures were carried out in *Escherichia coli* XL1-Blue or TOP10, using the Gibson assembly cloning method [68]. All constructs were verified using the GATC LIGHTRUN service (Eurofins Genomics, Ebersberg, Germany).

The plasmid pUAGC1160 was obtained by assembling F1 and F2 fragments with a linearized (SphI/BamHI) pUAGC280 vector. Fragment F1, comprising the *pipX* coding sequence and 141 bp upstream, was amplified by PCR from *S. elongatus* genomic DNA with primers CS3-PipX-1F/PipX-FL-1R. Fragment F2, comprising a 16 amino acid flexible linker (FL), as described in [19], and the coding sequence of SmBiT, was generated by PCR using plasmid pUAGC280 as a template and primers NSI-seq/FL-SmBiT-NS1-2F.

The plasmid pUAGC1161 was obtained by assembling F3 and F4 fragments with the linearized (BamHI) pUAGC1160 vector. Fragment F3, comprising the *glnB* coding sequence and 187bp upstream, was amplified by PCR from *S. elongatus* genomic DNA using primers FL-SmBiT-PII-3F/PII-FL-LgBiT-3R. Fragment F4, comprising an 8 amino acid FL, as described in [19], and the coding sequence of LgBiT, was generated by PCR using plasmid PII-ST-FL-LgBiT as a template and primers FL-LgBiT-4F/LgBiT-NS1-4R.

The plasmid pUAGC1163 was obtained by assembling fragments F5 with F6. Fragment F5, comprising the *ntcA* coding region and 195 bp upstream, was amplified by PCR from *S. elongatus* genomic DNA using primers SmBiT-PNtcA-F/NtcA-FL-LgBiT. Fragment F6 was amplified by PCR from pUAGC1161 and primers FL-LgBiT-4F/SmBiT-2R.

The plasmids pUAGC1162 and pUAGC1164 were obtained by Quickchange site-directed mutagenesis [69] with primers Y6A-2F/Y6A-2R, and pUAGC1161 and pUAGC1163 as templates, respectively.

### 3.2. Cyanobacterial Transformation and Strain Verification

Transformations were performed essentially as described in [70]. Verification of the correct inactivation of *pipX* or *pipXglnB* was confirmed by PCR analysis with oligonucleotide pairs PipX-126-F/PipX-5R and Glnb-1F/Glnb-1R, respectively. Verification of correct insertions at the NSI neutral site was confirmed by PCR analysis using the oligonucleotide pair NS1-1R/NS1-2R.

### 3.3. Cyanobacterial Growth and Culture Conditions

*S. elongatus* cultures were routinely grown in blue–green algae medium BG11 (BG11_0_ supplemented with 17.5 mM NaNO_3_ and 10 mM HEPES/NaOH, pH 7.8 [71]) at 30 °C under constant illumination provided by cool-white fluorescent lights, in flasks (70 μmol photons m^−2^s^−1^; shaking: 150 rpm) or on plates (50 μmol photons m^−2^s^−1^).

For solid media, 1.5% (*w*/*v*) agar and 0.5 mM sodium thiosulfate (Na_2_S_2_O_3_) were added after autoclaving. The required antibiotic/s were added at the following concentrations: chloramphenicol (Cm; 3.5 μg mL^−1^), streptomycin (Sm; 15 μg mL^−1^) or kanamycin (Km; 12 μg mL^−1^).

The growth of liquid cultures was monitored by measuring the optical density, at 750 nm (OD_750nm_), of 1 mL samples using an Ultrospec 2100 pro-UV-Vis Spectrophotometer (Amersham Biosciences, Amersham, UK). For experiments involving the transfer of cultures to media with different nitrogen sources, mid-exponential BG11 cultures were harvested by centrifugation (4500× *g*, 5 min), washed twice with BG11_0_ (no added nitrogen, -N) and resuspended in 20 mL of BG11, BG11_A_ (BG11_0_ supplemented with 10 mM NH_4_Cl and 10 mM HEPES/NaOH, pH 7.8), BG11_0_ or the same BG11 supernatant, at a final OD_750nm_ of 0.4.

### 3.4. Bioluminiscence Assays

To measure NanoBiT bioluminescence, 500 µL samples of cyanobacterial cultures were transferred to 3.5 mL luminometer tubes, mixed with 10 µL of freshly prepared Nano-Glo Live Cell Reagent (1:20 dilution of Nano-Glo Live Cell Substrate in Nano-Glo LCS Dilution Buffer; Promega Corporation, Madison, WI, USA) and incubated for 5 min under the same culture conditions (light, temperature and shaking). Their bioluminescence was quantified in a luminometer (Junior LB9509, Berthold Technologies GmbH & Co. KG, Bad Wildbad, Germany) using 10 s measuring times. Raw luminescence values were normalized using the OD_750_ of each culture and represented in the graphs as RLUs (relative luminescence units).

### 3.5. Intracellular ATP Content Determination

The ATP extraction was essentiality performed as described in [72]. Briefly, 500 µL aliquots were flash-frozen in liquid nitrogen. ATP was extracted via three consecutive cycles of boiling (100 °C) and freezing (liquid nitrogen), followed by centrifugation at 14,000× *g* for 1 min at 4 °C. A total of 50 µL of the supernatant was mixed with 50 µL BG11 and 40 µL of a reaction solution containing 1 mM DTT, 0.25 mM Luciferin and 75 µg/mL luciferase from *Photinus pyralis*. The bioluminescence was measured in black 96-well microplates (OptiPlate-96 F HB; PerkinElmer, Waltham, MA, USA) using a VICTOR3TM 1420 Multilabel Plate Reader (PerkinElmer, Waltham, MA, USA). An ATP standard curve was created in parallel. Each point was measured twice, the ATP content was quantified using the standard curve, and the data were normalized to the initial time 0 value.

### 3.6. Protein Extraction and Immunodetection

Samples of 10 mL from mid-exponential cultures (0.5–0.7 OD_750nm_) were harvested via 6 min centrifugation at 7300× *g* (4 °C) and stored at 20 °C. The pellets were resuspended in 60 μL of lysis buffer (25 mM Tris/HCl pH 7.5, 0.4 mM EDTA, 1 mM DTT, 0.8 mg/mL protease inhibitor, 50 mM NaCl), and cells were disrupted with 1 spoonful of 0.1 mm glass beads (≈30 µL), as described in [37]. Mixtures were subjected to three cycles of 60 s at a speed of 5 m/s in a high-speed homogenizer Minibeadbeater, followed by 60 s at 4 °C. Samples were centrifuged (5500× *g* for 5 min), and their supernatant fractions (crude protein extracts) were transferred to a new tube. Protein concentrations were estimated via the Bradford method using the PierceTM detergent-compatible Bradford assay kit (ThermoScientific, Waltham, MA, USA) on a VICTOR3TM 1420 Multilabel Plate Reader, and crude protein extracts were stored at −20 °C until needed.

For immunodetection, 60 µg of total protein extract was loaded into a sodium dodecyl sulphate polyacrylamide gel (SDS-PAGE; 15% polyacrylamide). Gel electrophoresis was followed by immunoblotting onto 0.1 μm polyvinylidene fluoride membranes (from GE Healthcare Technologies, Inc., Chicago, IL, USA), and the membranes were subsequently blocked with Tris-Buffered Saline (TBS-Tween; 20 mM Tris/HCl pH 7.5, 500 mM NaCl, Tween 20 0.1%) solution containing 5% non-fat dried milk for 1 h at room temperature and then incubated overnight in TBS-Tween with 2% non-fat dried milk with the corresponding primary antibody. Membranes were then incubated for 1.5 h at room temperature with a 1:150,000 dilution of ECL rabbit IgG and an HRP-linked F(ab’)2 fragment (from a donkey, GE Healthcare). The signal was detected using a SuperSignal WestFemto reagent (Thermo Fisher Scientific, Waltham, MA, USA) in a Biorad ChemiDoc Imager using the automatic exposure mode and avoiding pixel saturation. A 1:5000 dilution of primary anti-PipX, anti-PII or anti-PlmA antibodies was used separately. Western blot assays were performed for three independent clones of each strain.

### 3.7. Computational Methods

Protein intensity levels were quantified from the Western blot images using ImageJ software version 1.53 K. Bands were picked up using the “rectangle” function, and the area plot corresponding to their intensity was measured using the “wand” tool. Each area from the PipX and PII’s immunodetection was normalised using the corresponding area of PlmA and reference against the control WT strain.

A statistical Wilcoxon rank-sum test was performed using the RStudio program [73]. *p*-values were adjusted using the Holms–Bonferroni method.

## 4. Conclusions

The purpose of this work has been to gain insight into the complexities and idiosyncrasy of cyanobacterial signal transduction, exemplified here by the alternative and highly regulated association between PipX and its best-known binding partners, the signalling protein PII and the transcriptional regulator NtcA. We have used the NanoBiT complementation system to analyse the regulation of PipX-PII and PipX-NtcA complex formation in *S. elongatus*. To provide an intracellular environment that was as unperturbed as possible, NanoBiT gene fusions maintained their upstream regulatory regions while their endogenous counterparts, except for the essential *ntcA* gene, were deleted to prevent interference with the activity of the reporters.

The results obtained here match the wealth of existing information on PipX-PII and PipX-NtcA interactions, their effectors, the relative levels of these three proteins in *S. elongatus* and the effect of point mutations at PipX on these complexes. Importantly, they bring new light to the field by showing the exquisite sensitivity of the PipX-PII and PipX-NtcA complexes to specific changes and additional signals, suggesting the existence of an unanticipated negative feedback loop that tunes down the overactivation of NtcA by PipX and further revealing that PII has a transient role as a NtcA activator, stimulating PipX-NtcA complexes during their initial response to nitrogen deprivation.

In summary, this work expands our knowledge on the complexities of the PipX interaction network and provides, in a model cyanobacterium, proof of principle for a powerful tool to address the intricacies of signalling and interaction networks.

## Figures and Tables

**Table 2:** Strains.

Strain	Genotype, Relevant Characteristics	Reference or Source
*E. coli* XL1-Blue	*recA1 endA1 gyrA96 thi-1 hsdR17 supE44 relA1* lac [F ´ *proAB* lacI^q^*Z*∆*M15* Tn*10* (Tet^R^)]	[55]
*E. coli* TOP10	F^−^ *mcrA* Δ(*mrr-hsdRMS-mcrBC*) φ80*lacZ*ΔM15 *ΔlacX74 nupG recA1 araD139* Δ*(ara-leu)7697 galE15 galK16 rpsL* (Str^R^) *endA1* λ^−^	Invitrogen
WT	Wild-type *S. elongatus* PCC7942	Pasteur Culture Collection
*pipX*	*ΔpipX::cat*, Cm^R^	[38]
*pipXglnB*	*ΔpipX::cat glnB::CK.2*, Cm^R^ Km^R^	[33]
*pipX* 1^S^PipX-SmBiT	*ΔpipX::cat NSI::(P_pipX_:pipX:FL:SmBiT)*, Sm^R^ Cm^R^	This work
*pipXglnB* 1^S^PipX-SmBiT	*ΔpipX::cat glnB::CK2 NSI::(P_pipX_:pipX:FL:SmBiT)*, Sm^R^ Km^R^ Cm^R^	This work
*pipXglnB* 1^S^PipXSmBiT-PIILgBiT	*ΔpipX::cat glnB::CK2 NSI::(P_pipX_:pipX:FL:SmBiT P_glnB_:glnB:FL:LgBiT)*, Sm^R^ Cm^R^ Km^R^	This work
*pipXglnB* 1^S^PipX^Y6A^SmBiT-PIILgBiT	*ΔpipX::cat glnB::CK2 NSI::(P_pipX_:pipX^16ta>gc^:FL:SmBiT P_glnB_:glnB:FL:LgBiT)*, Sm^R^ Cm^R^ Km^R^	This work
*pipX* 1^S^PipXSmBiT-NtcALgBiT	*ΔpipX::cat NSI::(P_pipX_:pipX:FL:SmBiT P_ntcA_:ntcA:FL:LgBiT)*, Sm^R^ Cm^R^	This work
*pipX* 1^S^PipX^Y6A^SmBiT-NtcALgBiT	*ΔpipX::cat NSI::(P_pipX_:pipX^16ta>gc^:FL:SmBiT P_ntcA_:ntcA:FL:LgBiT)*, Sm^R^ Cm^R^	This work
*pipXglnB* 1^S^PipXSmBiT-NtcALgBiT	*ΔpipX::cat glnB::CK2 NSI::(P_pipX_:pipX:FL:SmBiT P_ntcA_:ntcA:FL:LgBiT)*, Sm^R^ Cm^R^ Km^R^	This work

## Data Availability

Data is contained within the article and Supplementary Material.

## Supplementary Materials

The following supporting information can be downloaded at https://www.mdpi.com/article/10.3390/ijms25094702/s1.
