# Supplementary material for: Analysing the Cyanobacterial PipX Interaction Network Using NanoBiT Complementation in Synechococcus elongatus PCC7942
Source: Int J Mol Sci. 2024 Apr 25;25(9):4702. doi: 10.3390/ijms25094702 (PMC11083307; doi:10.3390/ijms25094702)
Supplement: Supplementary file 1 [file ijms-25-04702-s001.zip › Table S1.pdf]

Table S1. Oligonucleotides

Name	Sequence (5′-3′)
CS3-PipX-1F	5' TTCTGTATGGAACGGGCATGCTGCTAAAAACTAGCCGCC 3'
PipX-FL-1R	5' TCCTCCGCCGCCAGAAGATCCAGAACCGGTCGCTGCGGCA GCAGATCTCAGAAAGGTTTGTTTGAAGACTTG 3'
NSI-seq	5' GGAGGCTTGGCAGACC 3'
FL-SmBit-NS1-2F	5' GATCTTCTGGCGGCGGAGGAATGGTCACCGGCTACCGGCTGTT CGAGGAAATCCTGTAGGATCCTCTAGAGTCGACCTC 3'
FL-SmBit-PII-3F	5' GATCTTCTGGCGGCGGAGGAATGGTCACCGGCTACCGGCTGTTCGAG GAAATCCTGTAGTAACAGGTTGCCTGTCTAGCC 3'
PII-FL-LgBit-3R	5' TCCGCTACTTCCGCCGCCTCCGCTGATTGCGTCGGCGTTTTTC 3'
FL-LgBit-4F	5' CGGAGGCGGCGGAAGTAGCGGAGTGTTTACCCTGGAAGATTTC 3'
LgBit-NS1-4R	5' GAGGTCGACTCTAGAGGATCTTAGCTGTTGATGGTCACTCT 3'
SmBiT-PNtcA-F	5' ATGGTCACCGGCTACCGGCTGTTCGAGGAAATCCTGTAGCCGCTGTTGGCTGCCTCC 3'
NtcA-FL-LgBit-R	5' AAACACTCCGCTACTTCCGCCGCCTCCGCTGCTGAACTGCTGACTCAGCGC 3'
SmBiT-2R	5' CTACAGGATTTCCTCGAACAGCCGGTAGCCGGTG 3'
Y6A-2F	5′ GCTTCCGAGAACGCCCTCAACCATCCC 3′
Y6A-2R	5′ CATTACTCGGGGGAATCG 3′
GlnB-1F	5' GGCTTAAGGAGAATTCCCTTGAAGAAG 3'
GlnB-1R	5' AACTGCAGTCGACGCTGACTTAGATTGCGTCG 3'
NS1-2R	5' CGGCCGAAAATGACAAGATC 3'
NSI-1R	5' TGCCTGAAAGCGTGACGAGC 3'
PipX-126-F	5' TAAAAACTAGCCGCCCTTGC 3'
PipX-5R	5' CAGCCCGCAAATCAGCAG 3'
