# Supplementary material for: Analysing the Cyanobacterial PipX Interaction Network Using NanoBiT Complementation in Synechococcus elongatus PCC7942
Source: Int J Mol Sci. 2024 Apr 25;25(9):4702. doi: 10.3390/ijms25094702 (PMC11083307; doi:10.3390/ijms25094702)
Supplement: Supplementary file 1 [file ijms-25-04702-s001.zip › Tables S1-S5.pdf]

**Table S2.** Bioluminescence values for the indicated strains and conditions. Number of biological replicates are indicated (X). nd = no data.

| | Time (min) | Nitrogen source | | |
|---|---|---|---|---|
| | | $NH_4^+$ | $NO_3^-$ | -N |
| PipX-PII (*pipXglnB*) | Pre-culture (5) | | 10190 ± 949 | |
| | 0 (5) | 20578 ± 5687 | 20423 ± 4903 | 21857 ± 3644 |
| | 15 (5) | 11376 ± 3927 | 9908 ± 3191 | 9166 ± 3052 |
| | 30 (1) | 14556 | 8565 | 3716 |
| | 45 (5) | 71172 ± 19042 | 57328 ± 26704 | 30305 ± 10466 |
| | 60 (5) | 80765 ± 21748 | 48768 ± 22756 | 19155 ± 13107 |
| | 120 (5) | 64529 ± 18988 | 34561 ± 18267 | 10025 ± 3702 |
| | 180 (5) | 46249 ± 14341 | 26102 ± 12850 | 8037 ± 3738 |
| | 240 (5) | 41024 ± 14048 | 26048 ± 8263 | 7448 ± 3418 |
| PipX-NtcA (*pipX*) | Pre-culture (3) | | 4811 ± 1364 | |
| | 0 (3) | 822 ± 50 | 671 ± 213 | 702 ± 208 |
| | 15 (3) | 783 ± 92 | 922 ± 322 | 753 ± 118 |
| | 30 | nd | nd | nd |
| | 45 (3) | 873 ± 116 | 2518 ± 408 | 2168 ± 727 |
| | 60 (3) | 610 ± 291 | 4029 ± 1134 | 5284 ± 877 |
| | 120 (3) | 733 ± 60 | 922 ± 58 | 3011 ± 1117 |
| | 180 (3) | 778 ± 160 | 1065 ± 239 | 3726 ± 1695 |
| | 240 (3) | 994 ± 277 | 1906 ± 434 | 3290 ± 1462 |
| PipX (*pipX*) | Pre-culture (3) | | 492 ± 134 | |
| | 0 (3) | 892 ± 507 | 1216 ± 354 | 1045 ± 429 |
| | 15 (3) | 945 ± 213 | 1128 ± 328 | 1071 ± 283 |
| | 30 | nd | nd | nd |
| | 45 (3) | 520 ± 151 | 956 ± 199 | 917 ± 406 |
| | 60 (3) | 640 ± 308 | 1037 ± 231 | 777 ± 441 |
| | 120 (3) | 844 ± 406 | 980 ± 308 | 804 ± 223 |
| | 180 (3) | 783 ± 238 | 1049 ± 381 | 565 ± 215 |
| | 240 (3) | 808 ± 194 | 1015 ± 332 | 612 ± 234 |
| PipX (*pipXglnB*) | Pre-culture (3) | | 404 ± 106 | |
| | 0 (3) | 742 ± 216 | 1046 ± 307 | 984 ± 293 |
| | 15 (3) | 693 ± 57 | 760 ± 25 | 802 ± 137 |
| | 30 | nd | nd | nd |
| | 45 (3) | 871 ± 108 | 885 ± 376 | 1012 ± 522 |
| | 60 (3) | 896 ± 121 | 828 ± 215 | 1259 ± 171 |
| | 120 (3) | 1334 ± 301 | 1128 ± 154 | 750 ± 6 |
| | 180 (3) | 787 ± 168 | 852 ± 208 | 863 ± 374 |
| | 240 (3) | 818 ± 78 | 960 ± 314 | 913 ± 3 |

**Table S3.** Bioluminescence values for the indicated strains and conditions. Number of biological replicates (2). nd = no data.

| | Time (min) | Nitrogen source | | |
|---|---|---|---|---|
| | | $NH_4^+$ | $NO_3^-$ | -N |
| PipX-NtcA (*pipX*) | Pre-culture | | 3429 ± 1017 | |
| | 0 | 956 ± 387 | 1025 ± 610 | 958 ± 249 |
| | 15 | 790 ± 8 | 1026 ± 387 | 856 ± 40 |
| | 30 | 3312 ± 3175 | 7312 ± 9008 | 9465 ± 1269 |
| | 45 | 5524 ± 146 | 10182 ± 5896 | 11421 ± 120 |
| | 60 | 3928 ± 4481 | 4572 ± 1691 | 8549 ± 5312 |
| | 120 | 3485 ± 1291 | 2393 ± 1075 | 4043 ± 760 |
| | 180 | 2600 ± 27 | 3730 ± 528 | 4468 ± 420 |
| | 240 | 4915 ± 241 | 2747 ± 381 | 2946 ± 964 |
| | 360 | 4853 ± 1882 | 10736 ± 3764 | 3091 ± 259 |
| PipX-NtcA (*pipXglnB*) | Pre-culture | | 4771 ± 440 | |
| | 0 | 690 ± 7 | 860 ± 159 | 712 ± 130 |
| | 15 | 958 ± 464 | 1271 ± 654 | 626 ± 40 |
| | 30 | 5283 ± 931 | 11537 ± 745 | 5190 ± 2632 |
| | 45 | 6759 ± 1802 | 8939 ± 3514 | 6183 ± 1961 |
| | 60 | 3892 ± 1657 | 4828 ± 996 | 9822 ± 4011 |
| | 120 | 4579 ± 925 | 1506 ± 1230 | 3564 ± 2592 |
| | 180 | 3546 ± 4143 | 2649 ± 1978 | 3533 ± 2578 |
| | 240 | 3318 ± 1164 | 3157 ± 2067 | 4538 ± 608 |
| | 360 | 2363 ± 1100 | 13437 ± 251 | 4415 ± 369 |

**Table S4.** Bioluminescence values for the indicated strains in the presence or absence of 200 μM DCCD. Number of biological replicates are indicated (X).

| | Time (min) | -DCCD | +DCCD |
|---|---|---|---|
| PipX-PII (*pipXglnB*) | 0 | 12755 ± 4028 | 12393 ± 2814 |
| | 5 | 10967 ± 2969 | 116980 ± 15137 |
| | 10 | 10057 ± 3919 | 149405 ± 16103 |
| | 15 | 9015 ± 3142 | 171105 ± 9677 |
| | 20 | 8956 ± 3010 | 121029 ± 38618 |
| PipX-NtcA (*pipX*) | 0 | 4402 ± 1451 | 4864 ± 865 |
| | 5 | 4262 ± 1793 | 1568 ± 509 |
| | 10 | 4311 ± 1966 | 657 ± 119 |
| | 15 | 4510 ± 1247 | 523 ± 40 |
| | 20 | 4770 ± 1451 | 400 ± 45 |

**Table S5.** Bioluminescence values for the indicated strains and conditions. Number of biological replicates are indicated (X). nd = no data.

| | Time (min) | Nitrogen source | | |
|---|---|---|---|---|
| | | $NH_4^+$ | $NO_3^-$ | -N |
| PipX-PII (*pipXglnB*) | 0 (2) | 24281 ± 7527 | 22837 ± 8400 | 23587 ± 5816 |
| | 60 (2) | 62194 ± 23344 | 27850 ± 12282 | 17449 ± 86 |
| PipX$^{Y6A}$-PII (*pipXglnB*) | 0 (3) | 1996 ± 627 | 2687 ± 1434 | 1653 ± 615 |
| | 60 (3) | 6374 ± 1476 | 2032 ± 1173 | 845 ± 189 |
| PipX-NtcA (*pipX*) | 0 (3) | 861 ± 35 | 673 ± 218 | 577 ± 139 |
| | 60 (3) | 4100 ± 2901 | 4592 ± 1805 | 8221 ± 4255 |
| PipX$^{Y6A}$-NtcA (*pipX*) | 0 (3) | 851 ± 96 | 681 ± 40 | 765 ± 83 |
| | 60 (3) | 710 ± 50 | 940 ± 204 | 867 ± 96 |
| PipX (*pipX*) | 0 (2) | 781 ± 38 | 899 ± 331 | 700 ± 107 |
| | 60 (2) | 433 ± 543 | 825 ± 338 | 1106 ± 567 |
