# Supplementary figures and images for: Analysing the Cyanobacterial PipX Interaction Network Using NanoBiT Complementation in Synechococcus elongatus PCC7942
Source: Int J Mol Sci. 2024 Apr 25;25(9):4702. doi: 10.3390/ijms25094702 (PMC11083307; doi:10.3390/ijms25094702)

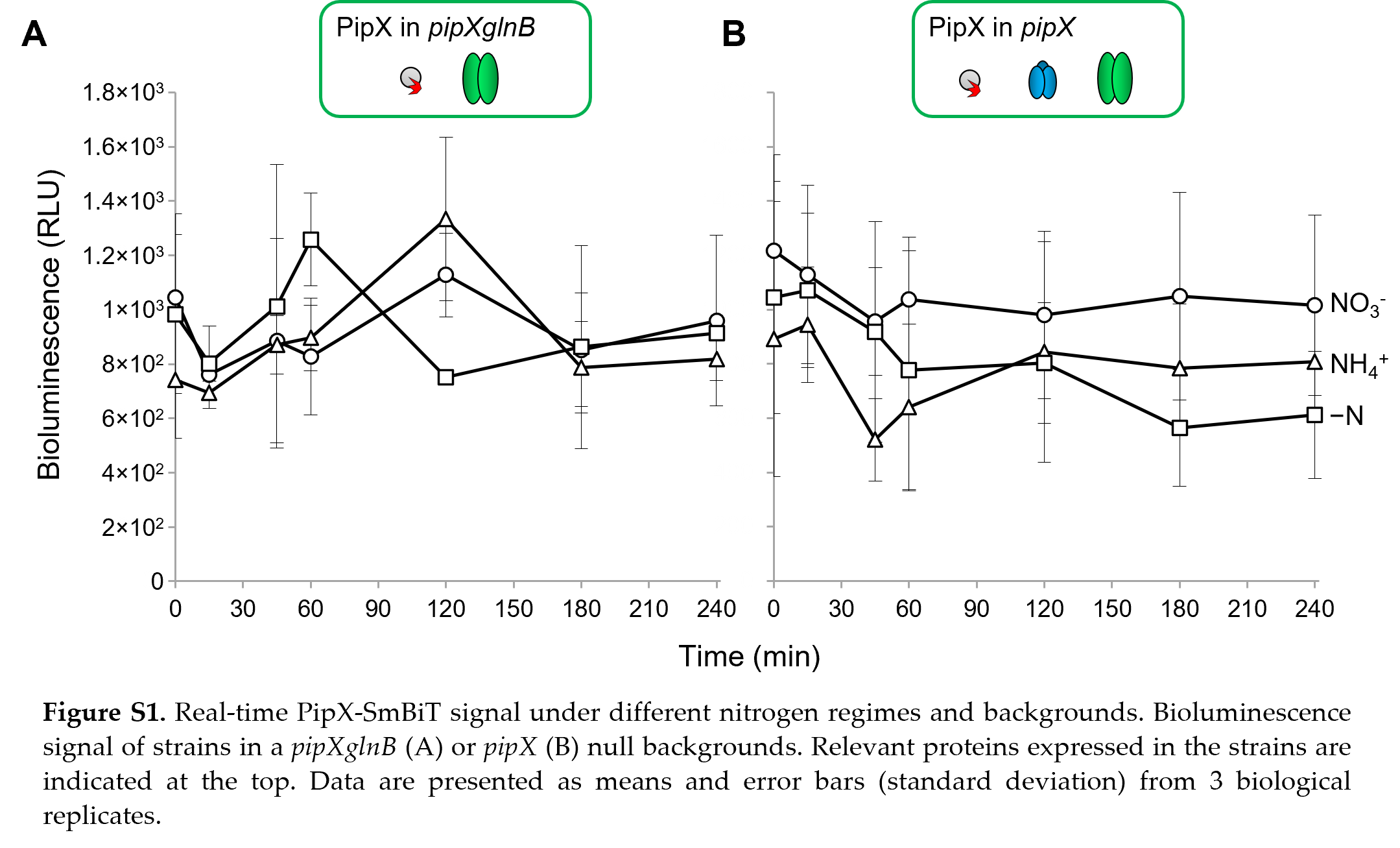

Supplement: Supplementary file 1 [file ijms-25-04702-s001.zip › Figure S1.tif]

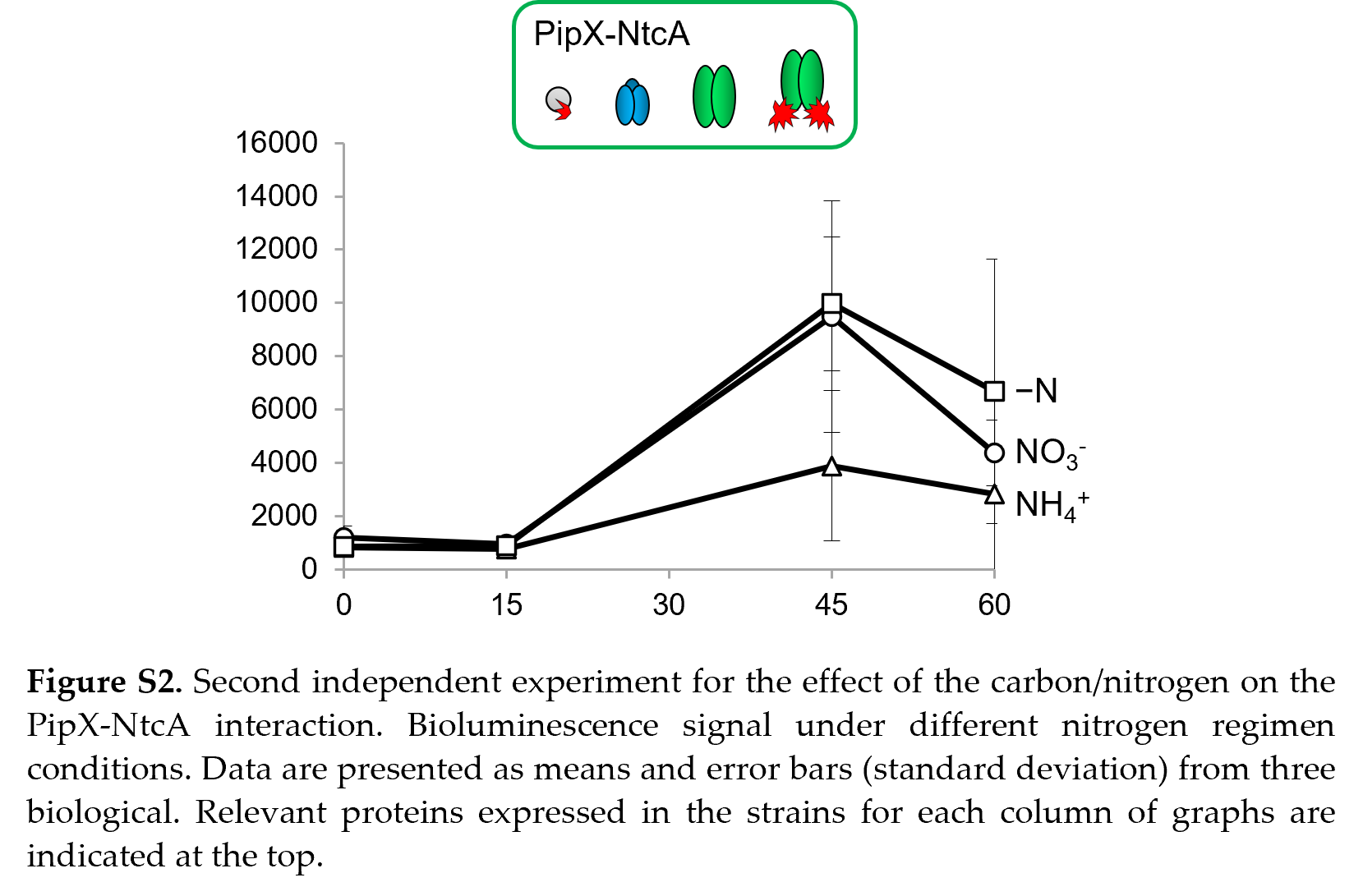

Supplement: Supplementary file 1 [file ijms-25-04702-s001.zip › Figure S2.tif]
